# The PTTG1-targeting miRNAs miR-329, miR-300, miR-381, and miR-655 inhibit pituitary tumor cell tumorigenesis and are involved in a p53/PTTG1 regulation feedback loop

**DOI:** 10.18632/oncotarget.5003

**Published:** 2015-08-07

**Authors:** Hai-qian Liang, Ren-jie Wang, Cai-feng Diao, Jian-wei Li, Jing-liang Su, Sai Zhang

**Affiliations:** ^1^ Department of Neurosurgery, Pingjin Hospital, Logistics College of Armed Police Forces, Tianjin, China, 300162; ^2^ Tianjin Saier-Biological Technology Company, Tianjin, China, 300353

**Keywords:** miRNAs, PTTG1, p53, pituitary tumor

## Abstract

Deregulation of the pituitary tumor transforming gene (PTTG1), a newly discovered oncogene, is a hallmark of various malignancies, including pituitary tumors. However, the mechanisms regulating PTTG1 expression are still needed to be explored. MicroRNAs (miRNAs) are a novel class of small RNA molecules that act as posttranscriptional regulators of gene expression and can play a significant role in tumor development. Here, we identified a series of miRNAs, namely, miR-329, miR-300, miR-381 and miR-655, which could target PTTG1 messenger RNA and inhibit its expression. Interestingly, all four miRNAs significantly that are downregulated in pituitary tumors were mapped to the 14q32.31 locus, which acts as a tumor suppressor in several cancers. Functional studies show that the PTTG1-targeting miRNAs inhibit proliferation, migration and invasion but induce apoptosis in GH3 and MMQ cells. Furthermore, overexpression of a PTTG1 expression vector lacking the 3′UTR partially reverses the tumor suppressive effects of these miRNAs. Next, we identified the promoter region of PTTG1-targeting miRNAs with binding sites for p53. In our hands, p53 transcriptionally activated the expression of these miRNAs in pituitary tumor cells. Finally, we found that PTTG1 could inhibit p53 transcriptional activity to the four miRNAs. These data indicate the existence of a feedback loop between PTTG1 targeting miRNAs, PTTG1 and p53 that promotes pituitary tumorigenesis. Together, these findings suggest that these PTTG1-targeting miRNAs are important players in the regulation of pituitary tumorigenesis and that these miRNAs may serve as valuable therapeutic targets for cancer treatment.

## INTRODUCTION

The pituitary tumor transforming gene (*PTTG1*), also known as securin, is a crucial component of the spindle checkpoint controlling faithful chromatid separation and has also been identified as a proto-oncogene [1, 2]. The PTTG1 protein is found at low levels in most normal adult tissues [3] but is over-expressed in various tumors, including pituitary [4, 5], lung [6], colorectal [7], and liver tumors [8]. PTTG1 plays a vital role in tumorigenesis. As the human securin, it ensures meticulous segregation of chromosomes during mitosis [2, 9], and both over- and underexpression of hPTTG causes genetic instability, resulting in tumor development [10–12]. In addition, hPTTG contributes to angiogenesis through transactivation of fibroblast growth factor (FGF)-2 and vascular endothelial growth factor (VEGF) via the Src homology 3 (SH3)-interacting domain [13–15]. As a transcriptional regulatory factor, PTTG1 exerts its transcriptional activity either by directly binding to DNA or by interacting with proteins, including PTTG1 binding factor, p53, Sp1, and upstream stimulatory factor 1 [16–18]. PTTG1 is regulated by miRNAs and other transcriptional activators [19–21]. These events promote the occurrence of tumors. Nevertheless, the upstream and downstream regulatory mechanisms of PTTG1 in pituitary tumors remain to be explored.

MicroRNAs (miRNAs) are a family of 21–25- nucleotide (nt) single-stranded non-coding RNA molecules. They can recognize target messenger RNA (mRNA) sequences at 3′-untranslated regions (3'-UTRs) by incomplete base-pairing, and they negatively regulate gene expression by mRNA destabilization or translational repression of target genes [22]. Recent studies have documented that miRNAs have important regulatory functions in biological processes that represent the hallmarks of cancer, such as proliferation, apoptosis, invasion, and metastasis [23]. miRNAs frequently form feedback loops because they are themselves regulated by transcription factors, which they directly or indirectly target [24, 25]. Such self-stabilizing circuits can be central components of epigenetic switches, where cellular phenotypes and expression patterns convert from one stable epigenetic state to another without changes in DNA sequence [26]. In the regulation of the downstream target genes, miRNAs may be affected by other regulators, such as p53 [27].

p53 is one of the most important tumor suppressor genes and is the most commonly mutated gene in human cancers [28]. The p53 protein is activated by a variety of cell stresses, such as DNA damage, hypoxia, inappropriate oncogene activation, spindle damage and hypoxia, resulting in an anti-proliferative response, including cell cycle arrest, apoptosis, or senescence [29]. The p53 stress-response pathway is heavily interconnected with miRNAs not only by regulating their expression and processing but also because p53 itself represents a down-stream target of miRNAs. In the last 5 years, the characterization of a number of miRNAs directly regulated by p53 and the cellular effects of these connections has been reported. p53 orchestrates such responses by directly activating key genes via binding two repeats of a diametric sequence with the RRRCWWGYYY consensus (where R stands for a purine, W for A/T and Y for a pyrimidine) p53-response element (p53-RE) [27, 30, 31]. In addition, some studies have reported that part of the transcription regulatory factors affect the function of p53. For example, PTTG1 (securin) specifically bound to p53 and interfered with DNA binding of p53, significantly reducing the transcriptional activity of p53 on p53-specific promoters [18].

In this study, we present evidence that PTTG1, behaving as a miR-329, miR-300, miR-381 and miR-655 target gene, acts to mediate cell transformation and tumor formation. We further investigate miRNA expression consistent with p53, which is accompanied by p53 binding to the miRNA promoters. Increasing PTTG inhibits p53 transcriptional activity by interaction. We uncovered a feedback circuit consisting of p53/miRNAs/PTTG1 and upregulation of PTTG1, which promotes tumorigenesis, thus providing a method to block pituitary tumor growth.

## RESULTS

### Identification of PTTG1-targeting miRNAs

**Figure 1:**
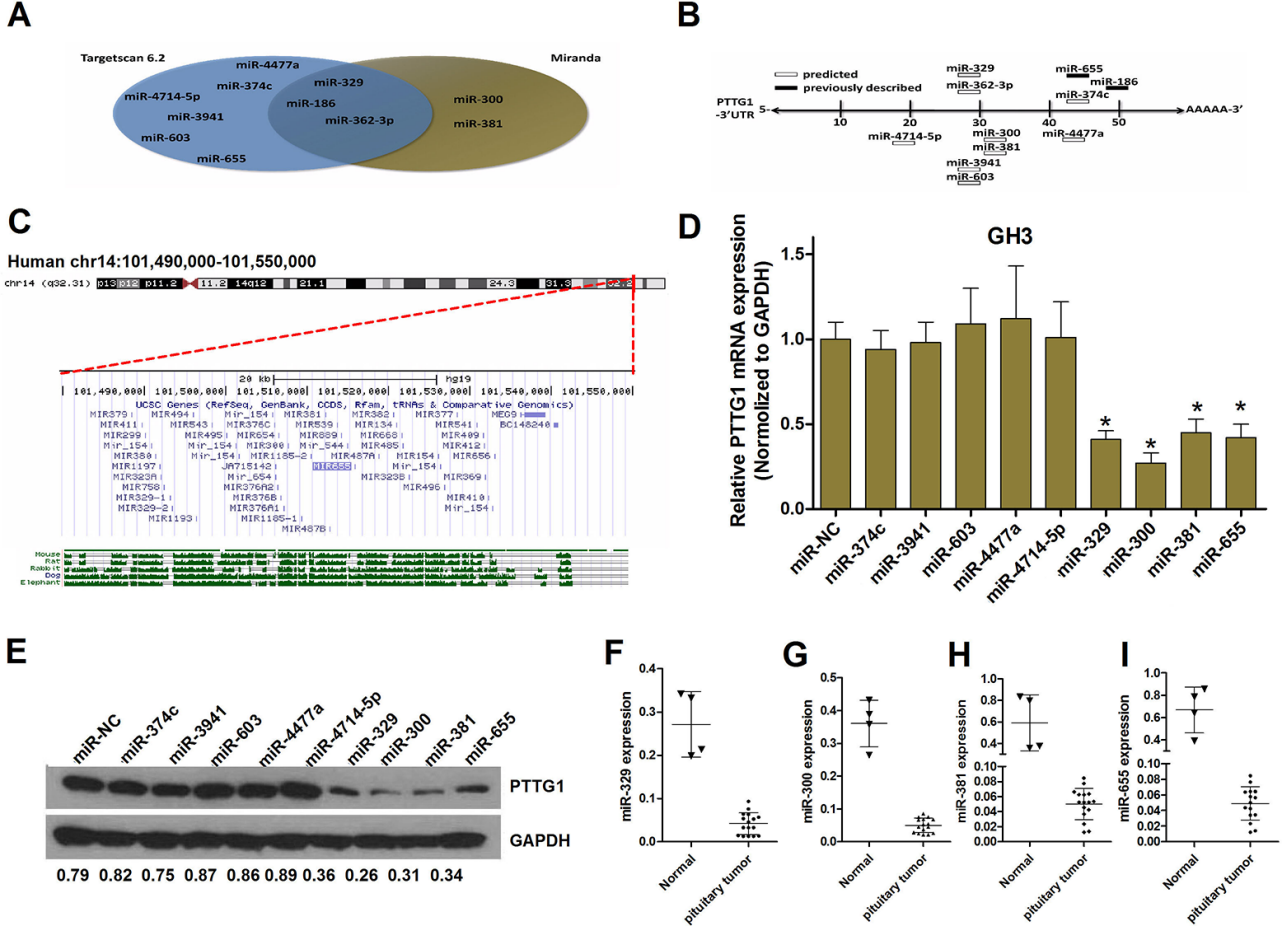
Identification of PTTG1-targeting miRNAs **A.** Potential miRNAs predicted to down-regulate the expression of PTTG1 (Targetscan6.2 and Miranda). **B.** MicroRNAs that target PTTG1 3′UTR, both predicted and previously described. **C.** A screenshot (http://genome.ucsc.edu; GRCh37/hg19 assembly) of the region of interest at the chromosomal level (red horizontal bar, top image) and in detail (bottom image), showing the 51 miRNAs located within 10 kb of each other. **D.** Real-time PCR analysis indicated that PTTG1 expression was significantly decreased in GH3 cells transfected with miRNA mimics (hsa-miR-NC, hsa-miR-374c, hsa-miR-3941, hsa-miR-603, hsa-miR-4477a, hsa-miR-4714-5p, hsa-miR-329, hsa-miR-300, hsa-miR-381, hsa-miR-655) (*P < 0.05). **E.** Measurement of PTTG1 expression levels by western blot analysis. Protein was extracted from GH3 cells transfected with miRNA mimics. The endogenous expression levels of the GAPDH protein were used for normalization, and the relative PTTG1 protein expression levels are shown. **F, G, H.** and **I.** Real-time PCR analysis indicated that miR-329, miR-300, miR-381, and miR-655 expression was significantly decreased in pituitary tumors relative to normal controls.

To identify miRNAs with the potential to down-regulate the expression of PTTG1, we used Targetscan6.2 and Miranda (Figure 1A). The results revealed that the miRNAs shown in Table 1 serve as a latent target, possessing conserved or poorly conserved target sites in the 3′UTR of PTTG1. Previously described and predicted PTTG1-targeting miRNAs are shown in Figure 1B. RNA hybridization was used to analyze the mfe mean between the miRNAs and the PTTG1 mRNA (Supplementary Figure S1). To our surprise, four of our predicted PTTG1-targeting miRNAs mapped to the common chromosomal 14q32.31 region, which contains a large cluster of intergenic miRNAs shown by others to be downregulated in tumors [32] (Figure 1C). To determine whether the predicted miRNAs could downregulate PTTG1, we transfected GH3 cells with miR-655, miR-186, miR-329, miR-381, miR-362-3p, miR-3941, miR-4477a, miR-4714, miR-374c or miR-603 mimics. Next, the PTTG1 mRNA and protein levels were detected by qRT-PCR and Western blotting. The expression of PTTG1 was markedly reduced after the ectopic expression of miR-655, miR-300, miR-381 and miR-329 in GH3 cells (Figure 1D and 1E). Because PTTG1 has been reported to be upregulated in pituitary tumor tissues, to confirm that the four miRNAs regulated PTTG1, we measured the expression of these miRNAs in pituitary tumor tissues. The results of qRT-PCR show that the levels of miR-655, miR-300, miR-381 and miR-329 are lower in sixteen pituitary tumor tissues compared to four normal pituitary glands (Figure 1F–1I). These data strongly suggest that miR-655, miR-300, miR-381 and miR-329 are regulated by PTTG1 and participate in PTTG1-mediated pituitary tumorigenesis.

**Table 1:** Identification of the PTTG1 targeting miRNAs

ID	Genome context	Predicted to target PTTG1 mRNA in its 3′UTR			Sequence
		RNAhybrid (mfe:kcal/mol)	miRanda (Yes/No)	Targetscan (Yes/No)	
hsa-miR-186	**chr1:** 71533314–71533399 [−]	−12.6	Yes	Yes	5′-C**AAAGAAU** UCUCCUUUUGGGCU-3′
hsa-miR-4477a	**chr9:** 68415308–68415388 [−]	−13.5	No	Yes	5′-C**UAUUAA** GGACAUUUGUGAUUC-3′
hsa-miR-603	**chr10:** 24564614–24564710 [+]	−15.9	No	Yes	5′-C**ACACAC** UGCAAUUACUUUUGC-3′
hsa-miR-3941	**chr10:** 124176481–124176583 [+]	−23.7	Yes	Yes	5′-U**UACACAC** AACUGAGGAUCAUA-3′
hsa-miR-655-3p	**chr14:** 101515887–101515983 [+]	−16.5	No	Yes	**5′-AUAAUAC AUGGUUAACCUCUUU-3′**
hsa-miR-329-3p	**chr14:** 101493122–101493201 [+]	−15.1	Yes	Yes	**5′-AACACA CCUGGUUAACCUCUUU-3′**
hsa-miR-381	**chr14:** 101512257–101512331 [+]	−20.8	Yes	No	**5′-UAUACAA GGGCAAGCUCUCUGU-3′**
hsa-miR-300	**chr14:** 101507700–101507782 [+]	−20.7	Yes	No	**5′- UAUACAA GGGCAGACUCUCUCU-3′**
hsa-miR-374c	**chrX:** 73438384–73438453 [+]	−18.1	No	Yes	5′-A**UAAUACA** ACCUGCUAAGUGCU-3′
has-miR-362-3p	**chrX:** 49773572–49773636 [+]	−18.6	Yes	Yes	5′-A**ACACAC** CUAUUCAAGGAUUCA-3′

### MiR-329, miR-300, miR-381 and miR-655 suppress cell proliferation activity and cell viability of GH3 and MMQ cells *in vitro* and *in vivo*

**Figure 2:**
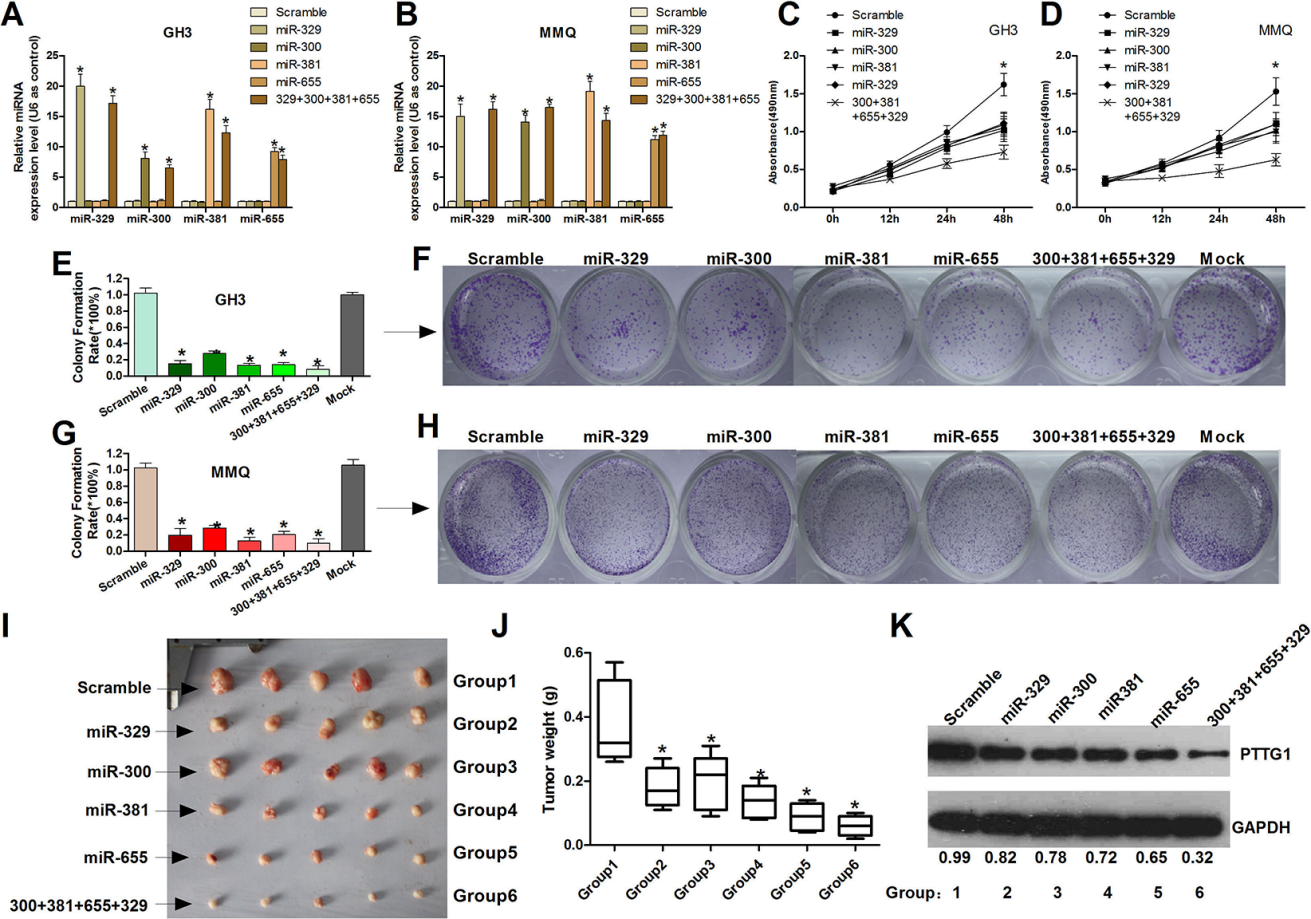
MiR-329, miR-300, miR-381 and miR-655 suppress cell proliferation activity and cell viability of GH3 and MMQ cells **A.** and **B.** Measurement of miR-329, miR-300, miR-381 and miR-655 expression levels by real-time RT-PCR. U6 snRNA served as an endogenous normalizer of expression. The relative miRNA expression levels (mean ± SEM) are shown (*P < 0.05). **C.** and **D.** Cell viability was detected using the MTT assay. The relative cell growth activity was normalized to the growth activity of GH3 and MMQ cells in the control groups (*P < 0.05). **E, F, G.** and **H.** MiR-329, miR-300, miR-381 and miR-655 inhibit GH3 and MMQ colony formation. GH3 and MMQ cells transfected with miR-329, miR-300, miR-381, and miR-655 mimics or scrambled were seeded in 12-well plates. On the 14th day after seeding, the number of colonies was counted. The quantitative results are shown as the mean ± SEM. The experiments were repeated three times (*P < 0.05). **I.** and **J.** The GH3 pituitary tumor growth *in vivo* was determined based on the tumor weight after euthanasia. A representative image of an *in vivo* tumor in each group is shown. Group 1–6 is the group of mice injected with Scramble, miR-329, miR-300, miR-381, miR-655 or mixed miRNAs. Each group contained 5 nude mice. The error bars represent the SEM. (*P < 0.05). **K.** The tumors in each group were collected and combined, and then digested for western blot analysis to detect PTTG1 expression.

To investigate the role of miR-329, miR-300, miR-381 and miR-655 in tumor cell proliferation and cell viability, miR-329, miR-300, miR-381 and miR-655 mimics were synthesized. The validation experiment was performed by qRT-PCR, which indicated a clear increase of these miRNAs in transfected GH3 and MMQ cells (Figure 2A and 2B). The MTT assay was used to characterize the effects of miR-329, miR-300, miR-381 and miR-655 on tumor cell viability. The results indicated that overexpression of miR-329, miR-300, miR-381 and miR-655 inhibited tumor cell viability (Figure 2C and 2D). In addition, a colony formation assay was used to demonstrate the anchorage-independent (long-term) growth effect of these miRNAs on GH3 and MMQ cells. The miR-329, miR-300, miR-381 and miR-655 mimics significantly decreased the long-term cell growth of pituitary tumor cells compared with the control groups (Figure 2E, 2F, 2G and 2H). These results demonstrate that miR-329, miR-300, miR-381 and miR-655 inhibit the viability and proliferation of human pituitary tumor cells.

To determine the effect of miR-329, miR-300, miR-381 and miR-655 on cell proliferation in GH3 cells *in vivo*, we treated GH3 xenograft tumor-bearing nude mice with a mimic control, miR-329 mimics, miR-300 mimics, miR-381 mimics and miR-655 mimics and the combined mimics (miR-381+miR-300 +miR-655+miR-329). Then, the tumor size and growth rate were measured. We found that miR-329, miR-300, miR-381 and miR-655 and the mixed miRNAs inhibited tumor growth compared to the control group (Figure 2I, 2J). In addition, we detected the expression of PTTG1 in each group of tumors. The Western blotting (Figure 2K) and immunohistochemical (Supplementary Figure S2) results show that PTTG1 decreased from group 1 to group 6, which suggests that these miRNAs inhibit pituitary tumor cell growth though regulating PTTG1.

### MiR-329, miR-300, miR-381 and miR-655 decrease cell motility *in vitro* and induce apoptosis in GH3 and MMQ cells

**Figure 3:**
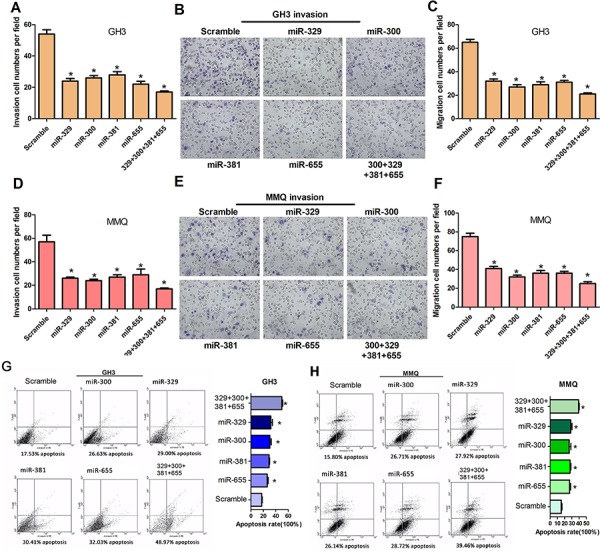
MiR-329, miR-300, miR-381 and miR-655 decrease cell motility *in vitro* and induce cell apoptosis of GH3 and MMQ cells **A–F.** Transwell assays were used to evaluate the migration and invasion of GH3 (A, B and C) and MMQ (D, E and F) cells transfected with miR-329, miR-300, miR-381, miR-655, miR-329+miR-300+miR-381+miR-655 or scrambled. Representative fields of invasive cells on the membrane. The data were drawn from three independent experiments (*P < 0.05). **G.** and **H.** Apoptosis of GH3 and MMQ cells after transfection with miR-329, miR-300, miR-381, miR-655, miR-329+miR-300+miR-381+miR-655 or scrambled were determined by flow cytometry. The incidence of apoptotic cells is shown. The cells were stained with annexin V-fluorescein isothiocyanate and counterstained with 7-ADD (*P < 0.05).

To determine whether miR-329, miR-300, miR-381 and miR-655 affect cell motility *in vitro*, cell invasion and migration assays were performed using transwell chambers with or without matrigel. GH3 and MMQ cells transfected with miR-329, miR-300, miR-381, miR-655 or mixed miRNA mimics were seeded in the transwell chambers and images were taken to count the cell numbers. The data revealed that miR-329, miR-300, miR-381 and miR-655 can suppress the ability of cells to migrate and invade relative to the control (Figure 3A, 3B, 3C, 3D, 3E and 3F). To further support the finding that miR-329, miR-300, miR-381 and miR-655 overexpression decreased the growth of GH3 and MMQ cells, FACS was used to analyze the apoptosis of miR-329, miR-300, miR-381 and miR-655-treated GH3 and MMQ cells. We observed that overexpression of miR-329, miR-300, miR-381 and miR-655 resulted in an increase in the cellular apoptosis rate compared to the negative control (Figure 3G and 3H).

### MiR-300, miR-381, miR-329 and miR-655 target PTTG1

**Figure 4:**
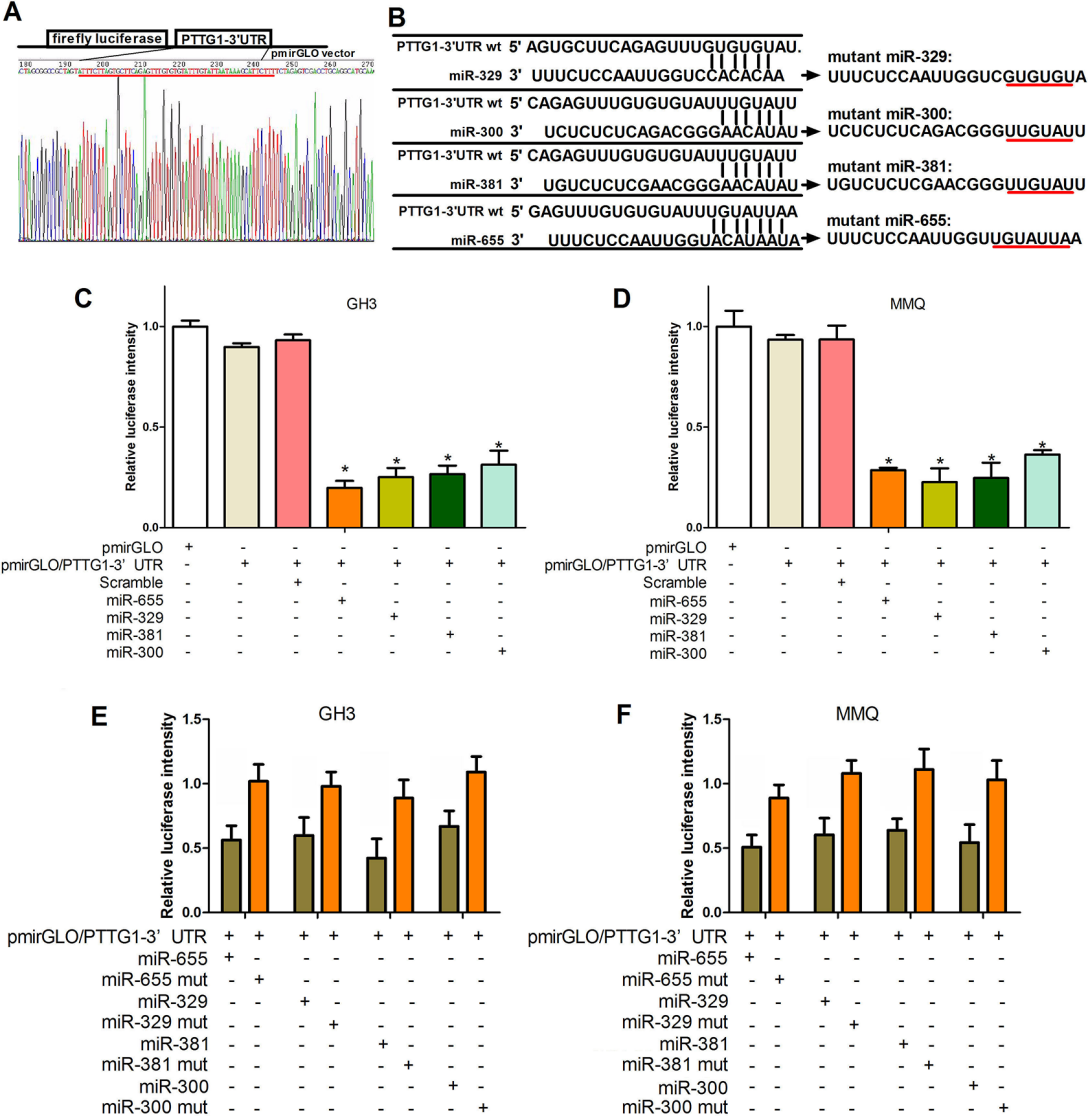
MiR-329, miR-300, miR-381 or miR-655 target PTTG1 **A.** The construction and sequencing map of PTTG1–3′UTR. **B.** The predicted miR-300, miR-381, miR-329 or miR-655 binding site on the PTTG1 mRNA 3′UTR and the mutation at the miRNA “seed region” binding site are shown. **C, D, E.** and **F.** GH3 and MMQ cells were transfected with the wild type version of the luciferase-PTTG1 3′-UTR reporter vector as well as the miR-300, miR-381, miR-329, or miR-655 mimics or scrambled. The miR-300, miR-381, miR-329, and miR-655 mimics reduced the intensity of the luciferase-PTTG1 3′-UTR reporter vector relative to the scrambled group, while the miR-300, miR-381, miR-329, and miR-655 mutants failed to alter the luciferase intensity. (*P < 0.05).

To elucidate whether the inhibition of pituitary tumor malignant behavior by the 14q32.31 miRNAs was mediated by PTTG1, we examined the interaction between miR-329, miR-300, miR-381 and miR-655 and the mRNA of PTTG1. We used a luciferase reporter system in which we cloned the PTTG1 3′-UTR fragments containing presumed target sites downstream of luciferase (Figure 4A). Subsequently, the potential mutant target sites of the miR-329, miR-300, miR-381 and miR-655 sequences were synthesized (Figure 4B). Co-transfection of a pmirGLO- reporter and miR-329, miR-300, miR-381 or miR-655 wild type mimics or mutants into GH3 and MMQ cells was undertaken. As shown in Figure 4C and 4D, the intensity of luciferase in GH3 and MMQ cells transfected with pmirGLO/PTTG1 3′-UTR and miR-329, miR-300, miR-381 and miR-655 mimics was lower than the control group. Importantly, miR-329, miR-300, miR-381 and miR-655 mutants did not affect luciferase intensity (Figure 4E and 4F). These results show that miR-329, miR-300, miR-381 and miR-655 regulate PTTG1 expression through direct binding of its 3′-UTR in GH3 and MMQ cells.

### PTTG1 overexpression counteracts mir-329, mir-300, mir-381 and mir-655

**Figure 5:**
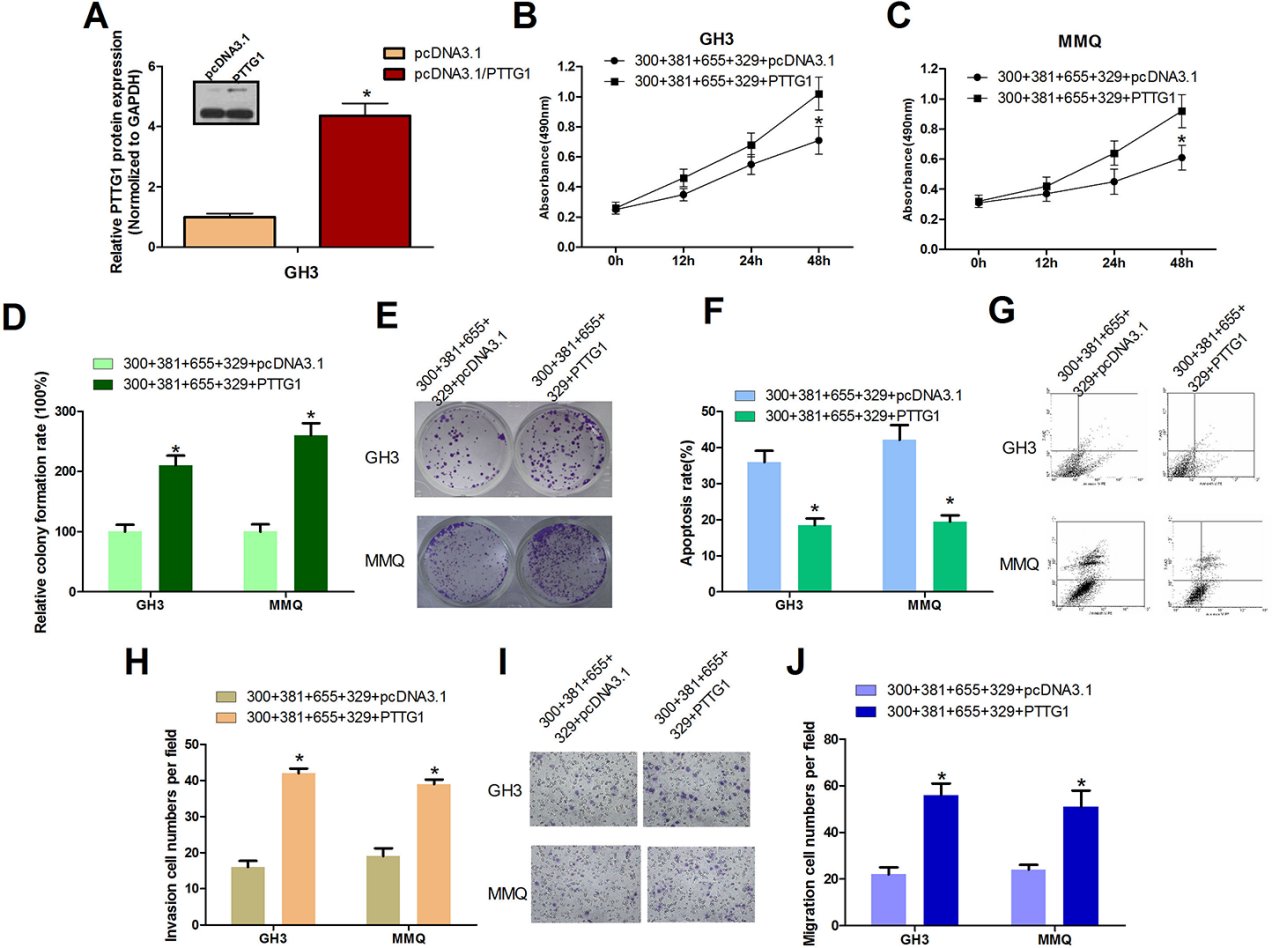
PTTG1 Overexpression Counteracts miR-329, miR-300, miR-381 and miR-655 induced pituitary tumor cell malignant inhibition **A.** Transfection with pcDNA3.1/PTTG1 increased PTTG1 protein levels in GH3 cells compared with the control group (*P < 0.05). **B.** and **C.** GH3 and MMQ cells were transfected with plasmids expressing PTTG1 or control vector, together with miR-329, miR-300, miR-381 and miR-655. Cell viability was detected by the MTT assay (*P < 0.05). **D.** and **E.** PTTG1 overexpression reversed the cell growth suppression caused by miR-300, miR-381, miR-655 and miR-329 in the colony formation assay. GH3 cells and MMQ cells were transfected with pcDNA3.1/PTTG1(PTTG1) or pcDNA3.1 with miR-300, miR-381, miR-655 and miR-329, and then seeded in 12-well plates. The colony formation rate is shown (*P < 0.05). **F.** and **G.** In GH3 and MMQ cells, PTTG1 ectopic expression reduced the cell apoptosis caused by miR-300, miR-381, miR-655 and miR-329. The cells were stained with annexin V-fluorescein isothiocyanate and counterstained with 7-ADD (*P < 0.05). **H–J.** Cell migration and invasion were increased by ectopic expression of PTTG1 in GH3 and MMQ cells (*P < 0.05).

To further investigate the role of PTTG1 in miR-329, miR-300, miR-381 and miR-655-mediated cell proliferation, cell viability, cell migration, cell invasion inhibition and cell apoptosis induction, we overexpressed PTTG1 by transfecting a construct (pcDNA3.1/PTTG1) that contains the PTTG1 ORF without its 3′UTR together with mixed miRNAs in GH3 and MMQ cells. The PTTG1 expression efficiency was measured (Figure 5A). Then, cell viability was measured using the MTT assay (Figure 5B, 5C); cell apoptosis (Figure 5F, 5G) was analyzed using FACS; cell proliferation was measured using a colony formation assay (Figure 5D, 5E); and cell invasion (Figure 5H, 5I) and migration assays (Figure 5J) were performed using transwell chambers with or without matrigel. We found that overexpression of PTTG1 partially mitigated the negative influence of PTTG1-targeting miRNAs on the progression of pituitary tumor cells.

### p53 binds the promoter of PTTG1-targeting miRNAs and induces miRNA expression

**Figure 6:**
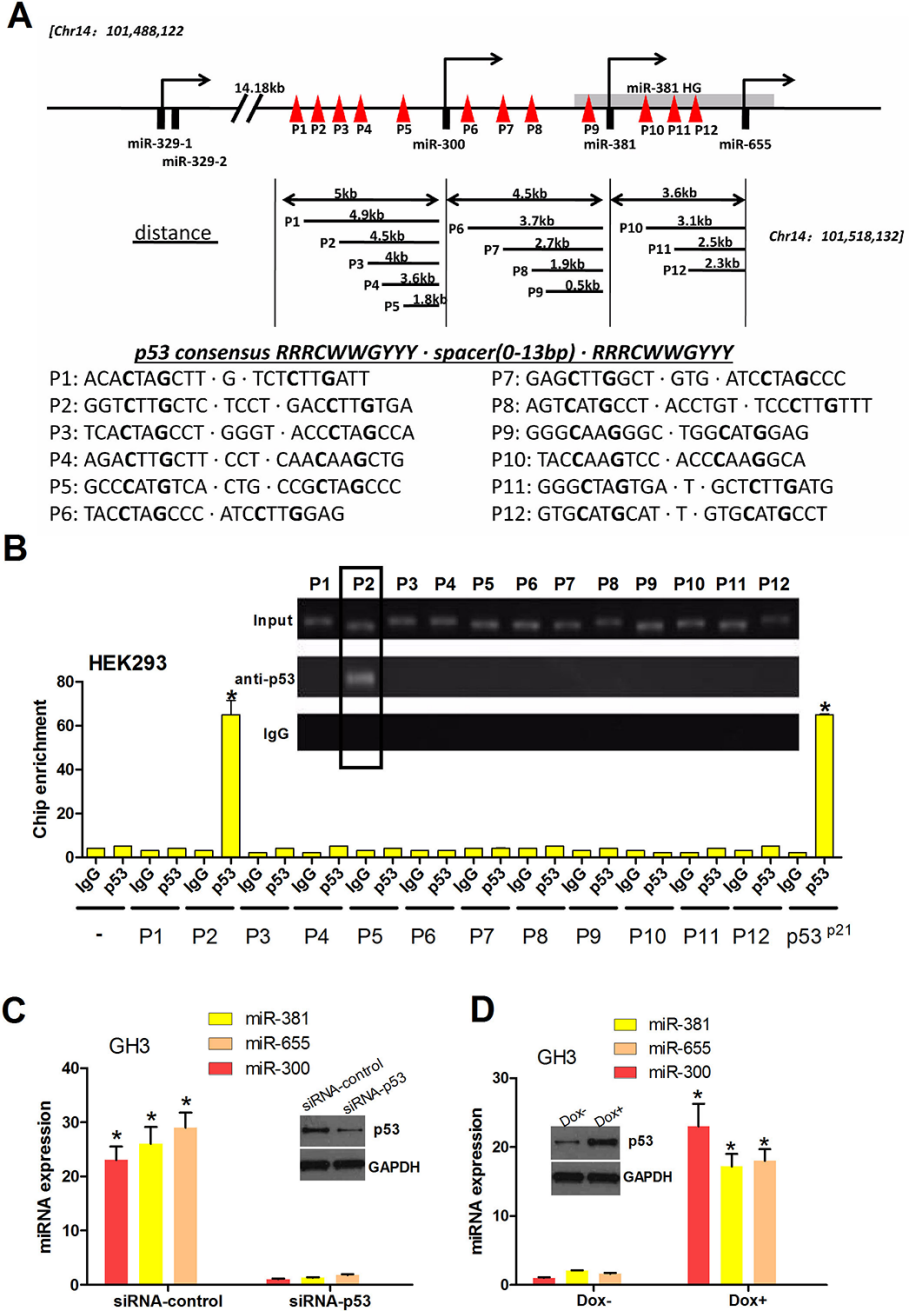
p53 binds the promoter of PTTG1-targeting miRNAs, activating miR-300, miR-381 and miR-655 transcription **A.** Schematic representation of potential p53 binding sites on the miRNA cluster promoter. Twelve predicted binding sites are shown. p53 is shown as a symbolic tetramer occupying a p53 binding motif containing two palindromic DNA sequences. P1-P12 represents the DNA-binding domain of p53 with PTTG1-targeting miRNAs. **B.** p53 binds the PTTG1-targeting miRNA promoter. Normalized inputs of HEK293 chromatin DNA were pulled down by p53 or negative IgG antibodies, and the DNA template was amplified by real-time PCR with specific PTTG1-targeting miRNA promoter primers (mentioned in Materials and Methods). A negative control primer was designed to assess the contribution of nonspecific binding. p53 binding sites in the p21 promoter were used as a positive control for the ChIP assay. Each negative IgG control was normalized to unit 1, each real-time PCR was performed in triplicate, and the ChIP experiments were repeated twice (*P < 0.05). **C.** p53 was reduced in GH3 cells treated with pSilencer2.1/p53-shRNA. Under this condition, PTTG1-targeting miRNAs were decreased in the GH3 cells. **D.** p53 was induced in GH3 cells treated with 1.0 mg/ml doxo for 16 h. Under this condition, PTTG1-targeting miRNAs were increased in the GH3 cells. Western blot analysis showed that p53 was reduced in GH3 cells treated with siRNA-p53 and induced in GH3 cells treated with 1.0 mg/ml doxo for 16 h.

As reported by others, p53 may play a vital role in regulating gene expression by directly activating the promoter region via binding two repeats of the DNA sequence, RRRCWWGYYY—NN—RRRCWWGYYY, including miRNA genes [30, 31]. We screened the human miR-300, miR-381 and miR-655 promoters with Genomatix MatInspector and detected 12 potential p53 binding sites (p53-Res), which we named P1-P12 (Figure 6A). Next, we performed chromatin immunoprecipitation (ChIP) to identify the p53 binding sites in the upstream region of the pri-miR-300, pri-miR-381 and pri-miR-655 genes. Equal amounts of sonicated HEK-293 chromatin DNA were incubated with the IgG control or p53 antibody. Protein G bead-captured chromatin DNA was amplified as template, and twelve pairs of primers were used for real time PCR (Figure 6B). Human p21 promoter primers were used as positive controls and α-satellite repeat primers as negative controls. The anti-p53 immunoprecipitated DNA was strongly amplified by P2, indicating specific p53 binding to the miR-300, miR-381 and miR-655 promoters around this region (Figure 6). To examine whether endogenous p53 promotes the expression of the PTTG1-targeting miRNAs in pituitary tumors, we attempted to reduce endogenous p53 using RNAi. The result shows that loss of p53 expression led to the downregulation of PTTG1-targeting miRNAs (miR-300, miR-381 and miR-655) (Figure 6C). Meanwhile, the tumor suppressor p53 can be activated by genotoxic stress, such as doxorubicin (dox). We treated the GH3 cells with doxo and found that gain of p53 led to the upregulation of these miRNAs (Figure 6D). These results demonstrate an important role for p53 in the induction of PTTG1-targeting miRNAs in pituitary tumor cells.

**Figure 7:**
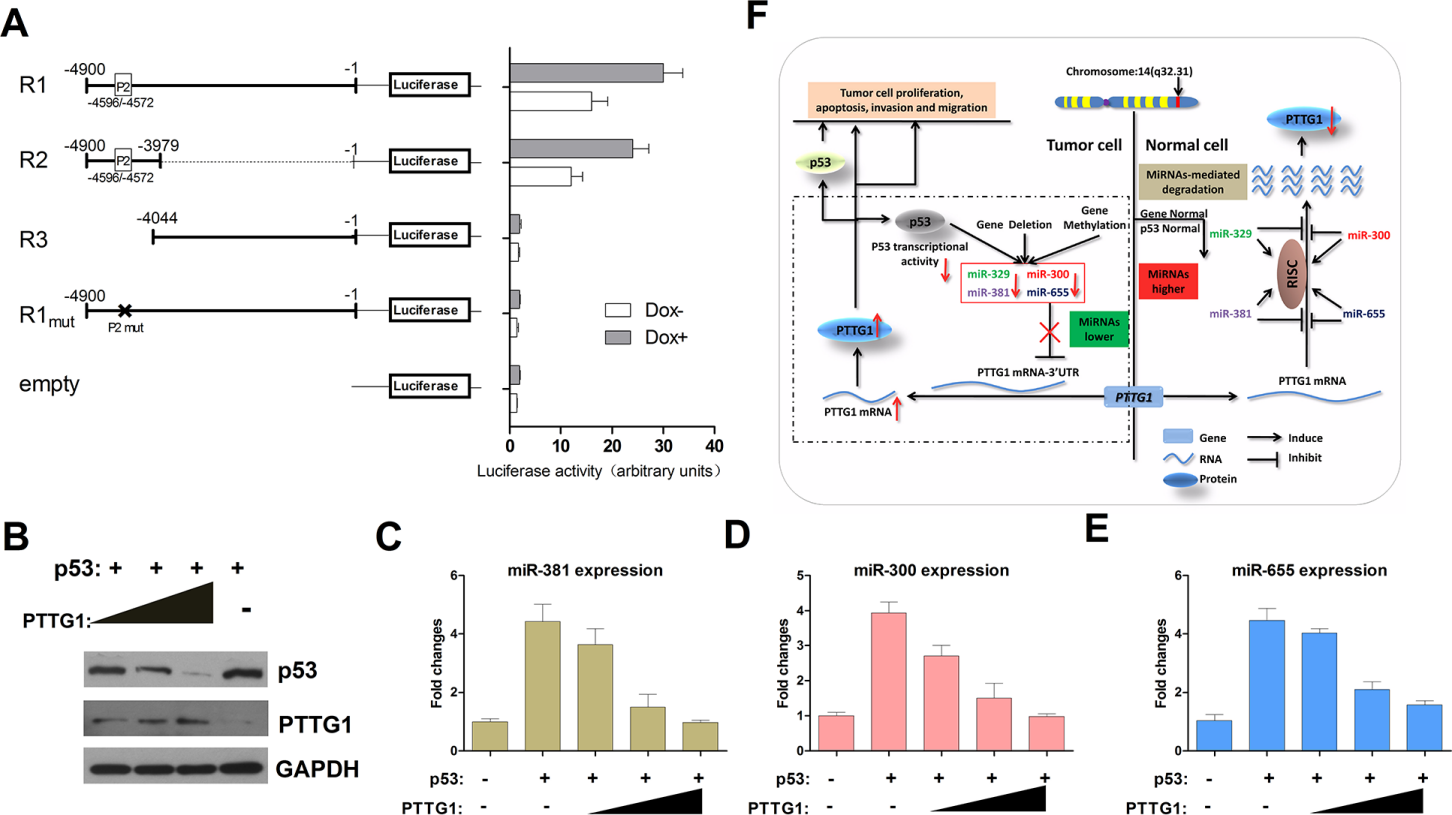
PTTG1, PTTG1-targeting miRNAs and p53 form a feedback loop **A.** Activity of the promoter constructs in Dox- and DOx+ HEK293 cells. The construct R1 indicates the full length from the position of the transcription start site (−1) to the upstream 4900 bp (−4900) regions containing the P2 p53 binding sites. Error bars represent standard deviations from three independent transfections each measured in triplicate. **B.** PTTG1 reduced p53 protein expression. GH3 cells were transfected with pcDNA3.1/PTTG1 and pcDNA3.1/p53. The p53 and PTTG1 protein was measured by western blotting. Western blots were quantified by Image-J and normalized to GAPDH. The experiments were repeated three times (*P < 0.05). **C, D.** and **E.** PTTG1 overexpression decreases the expression activity of miR-381, miR-300 and miR-655. GH3 cells transfected with pcDNA3.1/p53 together with increasing amounts of pcDNA3.1/PTTG1 (*P < 0.05). **F.** A schematic illustration of the proposed model depicting a feedback loop between PTTG1, PTTG1-targeting miRNAs and p53 that promotes pituitary tumor progression.

To further test for transcriptional activity, a series of genomic fragments was cloned into a promoterless luciferase reporter plasmid (Figure 7A). Reporter activity was then tested in Dox-treated (Dox+) and Dox-untreated (Dox-) HEK293 cells. The longest construct (R1) extended from 4.9 kb upstream of the most 5′ transcription start site and included the putative p53 binding site (Figure 6B, P2 -4596/-4572 bp). This fragment yielded robust p53-dependent transcriptional activity. Removal of this p53 binding site by truncation (R3) or mutation (R1_mut_) abolished transcriptional activity (Figure 7A). In addition, an 843 bp fragment containing the P2 binding site located upstream of the transcription start site (Figure 7A, R2) was sufficient for full promoter activity.

### PTTG1 modulates p53-dependent transcriptional activity of the PTTG1-targeting miRNAs

PTTG1 interacts with p53, blocking the specific binding of p53 to DNA and inhibiting its transcriptional activity [18]. A plasmid expressing p53, pcDNA3.1/p53, was cloned. We co-transfected pcDNA3.1/p53 with PTTG1, and western blotting revealed that p53 decreased gradually followed by an increase in PTTG1 (Figure 7B). Meanwhile, we tested PTTG1-targeting miRNA expression in transfected GH3 cells, and found that PTTG1-targeting miRNAs gradually decreased while PTTG1 expression increased (Figure 7C, 7D, 7E). These results demonstrate that p53 upregulates the activity of the miR-381 cluster promoter via p53 binding motifs and is inhibited by PTTG1. These data demonstrate that PTTG1-targeting miRNAs are positively regulated by p53, and this regulation was inhibited by PTTG1. A feedback loop exists between PTTG1, p53 and PTTG1-targeting miRNAs, which may contribute to pituitary tumor pathogenesis (Figure 7F).

## DISCUSSION

Here, we identified the presence of the PTTG1-targeting miRNAs/PTTG1/p53 feedback loop in GH3 and MMQ cells, suggesting that it may represent a new unifying mechanism of pituitary tumorigenesis. Several studies have reported that various tumors arise due to PTTG1 abnormal expression. In colorectal carcinogenesis, PTTG1 is a target of β-catenin transcriptional activation, which is overexpressed owing to loss-of-function mutations of the adenomatous polyposis coli (APC) tumor suppressor gene [19]. In addition, PTTG1 is overexpressed as an oncogene in pituitary [33–35], thyroid [15], kidney [36], breast [37], lung [38], and liver [39]. In our study, we reveal that PTTG1 is a direct target of miR-329, miR-381, miR-300 and miR-655, which may have an oncogenic role in miRNA-induced pituitary tumor progress inhibition. This result is consistent with previous studies; however, one report demonstrated that PTTG1 is a tumor suppressor. That study showed that in PTTG1-mutant females, the mammary epithelial cells showed increased proliferation and precocious branching morphogenesis. Additionally, mice lacking Pttg1 developed spontaneous mammary tumors. Moreover, in human breast tumors, PTTG1 protein levels were down-regulated and the reduction was significantly correlated with the tumor grade [40]. We suggest that the role of PTTG1 may be tissue specific.

Although a large number of miRNAs have been identified and implicated in many critical biological processes, including the progression of various human cancers, we further confirmed the regulatory mechanism of miRNAs, especially the miRNA clusters. In this study, ectopic expression of miR-329, miR-300, miR-381 and miR-655 suppressed cell proliferation activity and cell viability *in vitro* and *in vivo*, inhibited cell motility and induced cell apoptosis in GH3 and MMQ cells. As reported by others, miR-329 blocks the G1/S transition in LN18, dramatically suppresses cell proliferation and decreases colony formation [41]; miR-424 and miR-381 synergistically suppress the proliferation and survival of renal cancer cells [42]; and miR-655 up-regulation inhibits esophageal squamous cell carcinoma cell invasiveness by targeting PTTG1 [43]. We found that the four miRNAs were mapped to the 14q32.31 locus, which has been reported to act as a tumor suppressor in several cancers [32, 44, 45]. The expression of this cluster has been implicated in human malignancies including melanoma [46], ependymoma [47], neuroblastoma [48], hepatocellular carcinoma [49], and gliomas [50]. Recent observations show that 14q32.31 miRNAs regulate cellular behavior that is key to tumorigenicity. Our study strongly supports the hypothesis that the 14q32.31 locus acts as a tumor suppressor though its four members, miR-329, miR-300, miR-381 and miR-655, in pituitary tumors.

A number of studies have revealed deregulated miRNA levels in cancer, but whether such alterations directly cause tumorigenesis or simply result from changes in cellular phenotype remains unclear [51, 52]. In addition, transcriptional regulation, miRNA processing and maturation, and external stimuli all cause miRNA expression abnormities [24]. p53 can regulate miRNA directly or indirectly [27, 30]. The p53 transcription factor is encoded by a tumor suppressor gene, which is thought to be the most commonly mutated gene in human cancer [53]. We screened the human 14q32.31 locus cluster promoter with Genomatix MatInspector and detected several p53-binding sites. Accordingly, we performed chromatin immunoprecipitation (ChIP) to identify p53 binding to the PTTG1-targeting miRNAs promoter. In our hands, p53 activated miR-300, miR-381 and miR-655 transcription. Thus, the lack of transcriptional transactivation by p53, gene copy number deletion, and additional unknown mechanisms likely contribute to the loss of PTTG1-targeting miRNAs in pituitary tumors. Moreover, our study and others [54] indicated that PTTG1 could inhibit p53-dependent transcriptional activity of the PTTG1-targeting miRNAs.

In conclusion, we demonstrate that PTTG1-targeting miRNAs/PTTG1/p53 form a feedback loop in pituitary tumors, which may be a frequent event in diverse cancer subtypes. This circuit most likely can be triggered by extrinsic signals from the tumor microenvironment or cancer cell intrinsic signals (such as oncogene activation and tumor suppressor inactivation). While the constitutive activation of the components in the circuit is interdependent, each component regulates its own set of downstream genes that together drive cancer progression. Our results suggest that restoring PTTG1-targeting miRNAs using mimetics may have therapeutic potential for the treatment of pituitary tumors.

## MATERIALS AND METHODS

### Cell culture and transfection

The GH3 and MMQ cell lines were obtained from the China Infrastrure of Cell Line Resources (Beijing, China). GH3 was cultured in Dulbecco's modified Eagle's medium (DMEM) with high glucose (Hyclone Co., Logan, UT, USA) and 10% fetal bovine serum (Hyclone Co.). The MMQ and HEK293 cell lines were cultured in complete medium (F-12 supplemented with 15% horse serum and 2.5% FBS). All of the cell lines were grown at 37°C in a 5% CO_2_/95% air atmosphere. Cell transfection was performed in 70%–80% confluent cells using Lipofectamine 2000 Reagent (Invitrogen, USA) according to the manufacturer's protocol.

### Oligonucleotides

All of the RNA oligonucleotides were purchased from GenePharme (Shanghai, China). The miRNA sequences are listed in Table 1.

### RNA extraction and real-time PCR

For both mRNA and miRNA quantification, total RNA was extracted using the TRIZOL Reagent (Invitrogen). Five micrograms of total RNA was used to synthesize the first-strand cDNA with M-MLV (Invitrogen). Real-time PCR was amplified in 20 μl reaction mixtures using the following parameters: 95°C for 1 min, followed by 40 cycles of 95°C for 20 seconds and 56°C for 40 seconds. The 20 μl qPCR reaction consisted of 2X Platinum SYBR Green qPCR SuperMix UDG (Invitrogen, Life Technologies, Grand Island, NY, USA, 11733-046), 10 ng cDNA and 0.4 mM of forward and reverse primers. β-actin was used as the internal control. The specificity of the PCR reaction was monitored by a melt–curve protocol. For both mRNA and miRNA quantification, the data were imported into qBasePLUS (Biogazelle, Zwijnaarde, Belgium) for analysis of reference gene quality control and relative quantification. The primers used in this study are shown in Table 2.

**Table 2:** Primers used in the construction of plasmids and qRT-PCR

usage	name	sequence
qRT-PCR for PTTG1	PTTG1-S	5′-CAAACCCCTCCAACCAAAAG-3′
	PTTG1-AS	5′-CATCATCAGGAGCAGGAACA-3′
qRT-PCR for GAPDH	GAPDH-S	5′-CGTGACATTAAGGAGAAGCTG-3′
	GAPDH-AS	5′-CTAGAAGCATTTGCGGTGGAC-3′
Construction for PTTG1 3′UTR	PTTG1-UTR-S	5′-AAACTAGCGGCCGCTAGTATTTCTTAGTGCTTCA GAGTTTGTGTGTATTTGTATTAATAAAGCATTCTTTT-3′
	PTTG1-UTR-AS	5′-CTAGAAAAGAATGCTTTATTAATACAAATACA CACAAACTCTGAAGCACTAAGAAATACTAGCGGCCGCTAGTTT-3′
Construction for PTTG1 plasmid	PTTG1-S	5′-CGCGGATCCGCCACCATGGCTACTCTGATCTTTGTTG-3′
	PTTG1-AS	5′-CCGGAATTCTTAAATATCTGCATCGTAACAAAC-3′
Construction for R1 promoter	R1-S	5′-CAGCGAGCTCAAAGAATAGGGAGGACATAGG-3′
	R1-AS	5′-CGGAAGATCTAATGATGGCAGTGACAGGAAG-3′
Construction for R2 promoter	R2-S	5′-CAGCGAGCTCAAAGAATAGGGAGGACATAGG-3′
	R2-AS	5′-CGGAAGATCTCTGGGGTAGGTGTAGTAACC-3′
Construction for R3 promoter	R3-S	5′-CAGCGAGCTCTGGGCTGACCTTTCTCCCAAC-3′
	R3-AS	5′-CGGAAGATCTAATGATGGCAGTGACAGGAAG-3′
P53 bind site 1	P1-S	5′-TGAACAAAACCATGTGTAAC-3′
	P1-AS	5′-ATCAGTTTGCCTCCCATGTAG-3′
P53 bind site 2	P2-S	5′-TGAGTAGGTGGGACTACAG-3′
	P2-AS	5′-GTGGTGGGTGCCTGCAAT-3′
P53 bind site 3	P3-S	5′-TGCAGCCACATCCCACAG-3′
	P3-AS	5′-ACCAGCCAAGGAACTCTT-3′
P53 bind site 4	P4-S	5′-GCTGGTATGTGCAGAATGC-3′
	P4-AS	5′-TGCAGAGTTAGACATTCCT-3′
P53 bind site 5	P5-S	5′-CTCCCCCATGCGGAGAGT-3′
	P5-AS	5′-AGCATGAACCTAAGCACAATC-3′
P53 bind site 6	P6-S	5′-TACATGAAAGAATGACCGTC-3′
	P6-AS	5′-CAGCAGCAAGTCCTGTAGCC-3′
P53 bind site 7	P7-S	5′-CAAAGCCTCGGATGTCAGC-3′
	P7-AS	5′-CCATTAAACTCAGTGCAC-3′
P53 bind site 8	P8-S	5′-AGAAAGGTGATGGTTCCATT-3′
	P8-AS	5′-TGGAGCTGGGCCTGCACCT-3′
P53 bind site 9	P9-S	5′-GACCCTGTGCTCTTTCTTAG-3′
	P9-AS	5′-CAGGCACTGGATGAATTTACAC-3′
P53 bind site 10	P10-S	5′-CGGTCCACTAACCCTCAGCAT-3′
	P10-AS	5′-TACTGAAAAAGTGGATGACCCT-3′
P53 bind site 11	P11-S	5′-GTCCTTCATCGGGTATGG-3′
	P11-AS	5′-AGTGGAGCAAATGTTCTCG-3′
P53 bind site 12	P12-S	5′-TATGGCATCTTGCTTCCCT-3′
	P12-AS	5′-TCTCCTCAAGTATGAAACAG-3′

### Western blotting

Total cellular extracts were extracted using RIPA buffer. Proteins were separated by 10% SEMS–PAGE, and the proteins of interest were detected using the appropriate antibodies. Rabbit anti-human GAPDH antibody was from Saierbio (Tianjin, China). Mouse anti-human PTTG1 and rabbit anti-human p53 primary antibodies were obtained from Abcam (Cambridge, UK).

### Cell viability and proliferative capacity assay

The effect of miRNAs and the miRNAs /PTTG1 combination on the cell viability of GH3 and MMQ *in vitro* was assessed using the 3-(4,5-dimethylthiazol-2-yl)-2,5-diphenyltetrazolium bromide (MTT) cell viability assay. GH3 and MMQ cells were seeded in 96-well plates at a density of 5000 cells per 100 μl/well, and then transfected with miRNA mimics or scrambled on the next day. The MTT assay was used to determine relative cell viability at 0, 12, 24 and 48 h. Ten microliters of MTT (at a final concentration of 0.5 mg/ml) solution was added to 100 μl of culture medium and incubated for 4 h at 37°C. The absorbance at 490 nm (A490) was then measured using an uQuant Universal Microplate Spectrophotometer (Bio-Tek Instruments, USA).

The proliferative capacity was examined using colony formation assays. For the colony formation assay, the number of viable cell colonies was determined 15 days after the inoculation of 1000 cells/well in triplicate in 12-well plates. The cells were stained with crystal violet. The rate of colony formation was calculated using the equation: colony formation rate = (number of colonies/number of seeded cells) × 100%

### *In vitro* migration and invasion assays

For both migration and invasion assays, the cells were transfected with either a scrambled or a miRNA mimic. At 48 h post transfection, approximately 40,000 cells (migration) or 80,000 cells (invasion) were resuspended in FBS-free medium and seeded in a BD BioCoat control cell culture insert (BD Biosciences, San Jose, CA, USA) or a BD BioCoat matrigel invasion chamber (BD Bioscience, Catalog) for migration and invasion analysis, respectively. Note that migration/invasion inserts were placed in wells containing medium with 10% FBS in order to create a chemoattractive gradient. Twenty-four hours later, migrated/invaded cells were fixed, stained with crystal violet and counted using phase-contrast microscopy.

### *In vivo* assays

Athymic 6-week-old female nude mice were used for all *in vivo* experiments. Animal handling and procedures were performed according to a protocol approved by the Institutional Animal Care and Use Committee of Tianjin Medical University. GH3 cells (2 × 10^6^ cells) were injected into the right flank of nude mice. When tumor volumes reached 30–50 mm^3^, the mice were randomly assigned to groups (5 animals per group) and then treated with the mimic control, miR-381 mimics, miR-300 mimics, miR-655 mimics, miR-329 mimics or miR-381+miR-300 +miR-655+miR-329 mimics every three days. The tumor volumes were calculated using the following formula: (cubic millimeters) = (length × width^2^) × 0.5. Mouse weights were recorded every 2 days. We initiated the treatment at day 8 after implantation. After 21 days of treatment with the miRNAs, the mice were killed and the tumor tissues were collected.

### Potential miRNAs prediction and luciferase reporter assays

Based on bioinformatic prediction (TargetScan, RNAhybrid and http://microrna.org), miRNAs with the potential to target and down-regulate PTTG1 were selected (Table 1). The 3′UTR segments of PTTG1 containing putative binding sites for miRNAs were obtained by annealing and were inserted into the pmirGLO vector. The wild-type reporter construct pmirGLO/PTTG1–3′UTR was used for miRNA functional analysis, which was confirmed by DNA sequencing. All primer information is available in Table 2. For luciferase reporter experiments, GH3 and MMQ cells were co-transfected with pmirGLO/PTTG1–3′UTR reporter vector in a 48-well plate followed by the miRNA mimics or the mutant miRNAs. Firefly luciferase and Renilla luciferase levels were measured at 48 h after transfection. Each experiment was repeated at least three times.

### Promoter luciferase assay and constructs

The fragment of the miRNA promoter sequences containing the p53 target sites were obtained by PCR from the genome of HEK293. The acquired fragments were digested and ligated to a compatible pGL3-Basic vector (Promega). The cells were split into 24-well plates and each well was co-transfected with 200 ng luciferase vector pGL3-Basic as a negative control, pGL3 control vector as a positive control, or miRNA promoter, together with pSilencer/shR-p53. pRL-Tk (Promega) encoding Renilla luciferase was used as an internal control (5 ng/well) to assess transfection efficiency. After 48 hours, the whole-cell lysate was collected for reporter detection by the Dual Luciferase Reporter System (Promega). The reactions were measured using an Orion Microplate Luminometer (Berthold Detection System). The transfections were performed in triplicate, and repeated three times to assure reproducibility.

### Plasmid construction

To construct the PTTG1 expression vector pcDNA3.1/PTTG1, a 600 bp fragment was amplified by PCR using Rattus cDNA as a template. The primer sequences are listed in Table 2. The siRNA expression vectors pSilencer/shR-p53 and pSilencer/shR-PTTG1 were a gift of Tianjin Saier-Biological Technology Company.

### Chromatin immunoprecipitation (ChIP)

Ten million cells were cross-linked and lysed using the ChIP-IT express kit (Active Motif, Carlsbad, CA). Chromatin was sonicated to 200–800 bp length fragments with eight rounds of 10-second pulses using 25% power. Normalized inputs of sheared chromatin DNA were incubated with 4 μg negative control IgG and p53 (Abcam #ab179477) overnight at 4°C. The PCR reactions were amplified using precipitated immunocomplexes as the template. The miRNAs promoter primers are listed in Table 2. Human c-Fos promoter primers were used as positive controls (Cell Signaling #4663) and α-satellite repeat primers (Cell Signaling #4486) as negative controls.

### Statistical analysis

The results are expressed as the mean ± standard deviation (SEM) of values obtained in at least three independent experiments. Differences between samples were analyzed by Student's *t*-test. Differences reaching a *P* value of 0.05 were considered significant. All calculations were performed using the 14.0 SPSS software package (SPSS Inc., Chicago, IL, USA).

## SUPPLEMENTARY FIGURES


