# Supplementary material for: The PTTG1-targeting miRNAs miR-329, miR-300, miR-381, and miR-655 inhibit pituitary tumor cell tumorigenesis and are involved in a p53/PTTG1 regulation feedback loop
Source: Oncotarget. 2015 Aug 7;6(30):29413–27. doi: 10.18632/oncotarget.5003 (PMC4745736; doi:10.18632/oncotarget.5003)
Supplement: Supplementary file 1 [file oncotarget-06-29413-s001.pdf]

SUPPLEMENTARY FIGURES

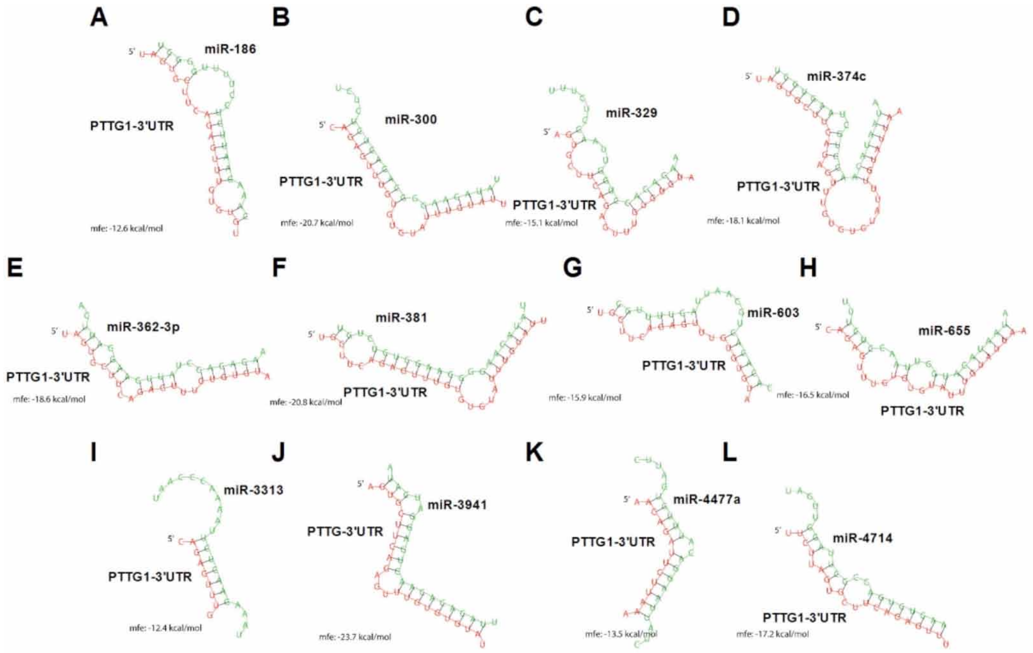

Supplementary Figure S1: RNAhybrid was used to analysis the mfe mean between the miRNAs and the PTTG1 mRNA. RNA-hybrid(http://bibiserv.techfak.uni-bielefeld.de/rnahybrid) was used to analysis the mfe mean between the PTTG1-targeting miRNAs and PTTG1 mRNA. As shown in Figure S1, the low mean of the mfe in B,C,F and H indicates miR-300, miR-381, miR-329 and miR-655 potentially directly interacted with PTTG1 mRNA.

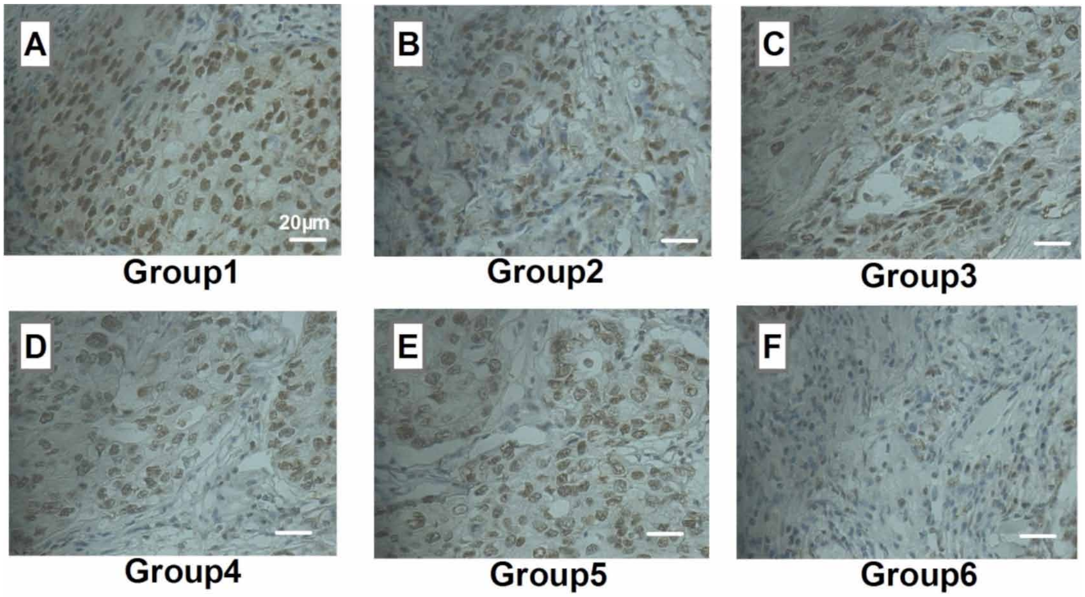

G

PTTG1 immunohistochemical staining

Scores	0	1	2	3	4
Group1	0	0	0	0	7
Group2	0	0	3	2	2
Group3	0	1	3	2	1
Group4	0	0	2	3	2
Group5	0	1	1	4	1
Group6	1	6	0	0	0

Supplementary Figure S2: PTTG1 was inhibits by PTTG1-targeting miRNAs in nude mice model. A–F. Immunohistochemical staining of PTTG1 in GH3 xenograft tumor-bearing nude mice transfected with scramble, miR-300, miR-329, miR-381, miR-655 or their collection. (A)Scramble, score 4; (B) miR-329-Group1, score 3; (C) miR-300-Group2, score 3; (D) miR-381-Group3, score 3; (E) miR-655-Group4, score 3; (F)PTTG1-targeting miRNAs plus-Group6, score 1. **G.** The cases of each score were counted and are shown in the table.
